# Regional Features of Long-Term Exposure to PM_2.5_ Air Quality over Asia under SSP Scenarios Based on CMIP6 Models

**DOI:** 10.3390/ijerph18136817

**Published:** 2021-06-25

**Authors:** Sungbo Shim, Hyunmin Sung, Sanghoon Kwon, Jisun Kim, Jaehee Lee, Minah Sun, Jaeyoung Song, Jongchul Ha, Younghwa Byun, Yeonhee Kim, Steven T. Turnock, David S. Stevenson, Robert J. Allen, Fiona M. O’Connor, Joao C. Teixeira, Jonny Williams, Ben Johnson, James Keeble, Jane Mulcahy, Guang Zeng

**Affiliations:** 1 Innovative Meteorological Research Department, National Institute of Meteorological Sciences, Seogwipo-si 63568, Jeju-do, Korea; sunghm122@korea.kr (H.S.); skysh2002@korea.kr (S.K.); jskim326@korea.kr (J.K.); jhlee0@korea.kr (J.L.); masun@korea.kr (M.S.); hellbent@korea.kr (J.S.); bellfe@korea.kr (J.H.); yhbyun@korea.kr (Y.B.); kyh@korea.kr (Y.K.); 2 Met Office Hadley Centre, Exeter EX1 3PB, UK; steven.turnock@metoffice.gov.uk (S.T.T.); fiona.oconnor@metoffice.gov.uk (F.M.O.); joao.teixeira@metoffice.gov.uk (J.C.T.); ben.johnson@metoffice.gov.uk (B.J.); jane.mulcahy@metoffice.gov.uk (J.M.); 3 University of Leeds Met Office Strategic (LUMOS) Research Group, School of Earth and Environment, University of Leeds, Leeds LS2 9JT, UK; 4 School of GeoSciences, The University of Edinburgh, Edinburgh EH9 3FF, UK; david.s.stevenson@ed.ac.uk; 5 Department of Earth and Planetary Sciences, University of California Riverside, Riverside, CA 92521, USA; rjallen@ucr.edu; 6 National Institute for Water and Atmospheric Research, Wellington 6022, New Zealand; jonny.williams@niwa.co.nz (J.W.); guang.zeng@niwa.co.nz (G.Z.); 7 Department of Chemistry, University of Cambridge, Cambridge CB2 1TN, UK; jmk64@cam.ac.uk; 8 National Centre for Atmospheric Science, University of Cambridge, Cambridge CB2 1EW, UK

**Keywords:** CMIP6, SSP scenarios, PM_2.5_, air quality index, Asia, climate changes

## Abstract

This study investigates changes in fine particulate matter (PM_2.5_) concentration and air-quality index (AQI) in Asia using nine different Coupled Model Inter-Comparison Project 6 (CMIP6) climate model ensembles from historical and future scenarios under shared socioeconomic pathways (SSPs). The results indicated that the estimated present-day PM_2.5_ concentrations were comparable to satellite-derived data. Overall, the PM_2.5_ concentrations of the analyzed regions exceeded the WHO air-quality guidelines, particularly in East Asia and South Asia. In future SSP scenarios that consider the implementation of significant air-quality controls (SSP1-2.6, SSP5-8.5) and medium air-quality controls (SSP2-4.5), the annual PM_2.5_ levels were predicted to substantially reduce (by 46% to around 66% of the present-day levels) in East Asia, resulting in a significant improvement in the AQI values in the mid-future. Conversely, weak air pollution controls considered in the SSP3-7.0 scenario resulted in poor AQI values in China and India. Moreover, a predicted increase in the percentage of aged populations (>65 years) in these regions, coupled with high AQI values, may increase the risk of premature deaths in the future. This study also examined the regional impact of PM_2.5_ mitigations on downward shortwave energy and surface air temperature. Our results revealed that, although significant air pollution controls can reduce long-term exposure to PM_2.5_, it may also contribute to the warming of near- and mid-future climates.

## 1. Introduction

Air pollution has now emerged as a leading global environmental health risk factor. In particular, long-term exposure to fine particulate matter, referred to as aerosols, is associated with increased rates of chronic bronchitis, reduced lung function, and increased mortality from lung cancer and heart disease [1]. The World Health Organization (WHO) reports that 92% of the world’s population resides in areas where the air-quality levels exceed the WHO’s ambient air-quality guidelines for the annual mean of particulate matter with a diameter of less than 2.5 μm (PM_2.5_) [2]. Moreover, approximately three million premature deaths occur annually as a result of long-term exposure to ambient air pollution [2,3]. Significantly high anthropogenic aerosol concentrations, especially recently in Asian regions, are linked to population growth and rapid industrialization. A recent report by the Organization for Economic Co–operation and Development (OECD) determined that South Korea, China, and India will endure the most significant economic damage and highest premature death rates in 2060 due to air pollution [4]. In addition, aerosols influence global and regional climate characteristics by altering the Earth’s radiative balance [5,6,7]. Previous studies [8,9,10] estimated that the present-day effective radiative forcing (ERF) by total aerosols is approximately −1 Wm^−2^, masking a considerable fraction of the well-mixed greenhouse gases (GHGs) induced by warming since the pre-industrial period.

Extensive research in recent decades has confirmed that the increase in surface PM_2.5_ concentrations in Asian regions, most notably since the middle 20th century, has been caused by the emission of primary aerosols and their precursors from industrial sources [7,11,12,13]. However, recent substantial efforts by the Chinese and Korean governments to mitigate anthropogenic emissions have resulted in a gradual decrease in the PM_2.5_ concentrations over East Asia [14,15]. Despite these efforts, current air pollution levels in Asian countries remain substantially higher than those of the United States and Europe. According to previous studies [16,17,18,19,20,21], the main cause of premature death in East Asia in the future will be the long-term exposure to elevated concentrations of ambient PM_2.5__._ Moreover, regional changes in PM_2.5_ concentrations are greatly affected by local emissions and the long/distant transport of aerosols [22,23,24]. In addition, significant temperature variations in Asia during the 20th century may have been related to large regional historic anthropogenic aerosol emissions and their radiative effects, which are more regionally confined than those of GHGs due to their relatively short lifetimes [6,13]. Because of the widespread environmental impacts such as visibility impairment and severe threats posed to human health, understanding the effects of fine particulate matter on human health and climate interactions have become critical to regional policymakers.

Recent chemistry–climate modeling studies have attempted to account for the geographical variations in aerosol emissions and transportation and consider the effect of these factors on the climate system [25,26,27,28]. In addition, experiments performed as part of the Coupled Model Intercomparison Project Phase 6 (CMIP6) [29] and the Aerosol Chemistry Model Intercomparison Project (AerChemMIP) [30] have contributed significantly to the multimodel (including interactive tropospheric chemistry and aerosols) evaluation of historical and future changes in air pollutants [10,31,32,33,34]. Aerosol concentrations are expected to reduce globally in the future, albeit at different paces over different regions, with reductions initially expected to occur in developed countries in Western Europe and North America. However, Asia, the world’s most populated and rapidly developing region, has been the largest source of aerosols and their precursors over the last few decades [35,36]. Therefore, the changes in regional aerosol concentrations caused by future air pollution mitigation could have important consequences for human health and climate change in the global and Asian countries.

This study aimed to assess the annual mean surface PM_2.5_ concentration and air quality in Asian regions (East Asia, South Asia, and Southeast Asia) and conduct future projections using simulations from CMIP6 multimodel ensembles. Future scenarios have been developed for the Scenario Model Intercomparison Project (ScenarioMIP) of CMIP6: the shared socioeconomic pathways (SSP), which were combined with the Representative Concentration Pathways (RCP) [29]. The qualitative and quantitative aspects of these new scenarios have been extensively discussed in the literature [37,38,39,40]. In the SSP scenarios, the level and speed of air pollution controls in each region are implemented differently. While SSP3 (regional rivalry) and SSP4 (inequality) assume the slowest deployment of air pollution controls, SSP1 (sustainability) and SSP5 (conventional development) adopt the fastest and widest implementations, as shown in Table 1 [41,42]. Moreover, the SSP2 scenario implements significant advances in air pollution control. Air pollution controls in the SSP scenarios depend on the income levels of the countries. Therefore, each country in the Asian region will implement air-pollution controls with various schedules according to their individual institutional, financial, and technological capacities. Detailed information on air pollution control per country according to the SSP scenarios can be found in Rao et al. [43]. This study was designed to explore the future changes in PM_2.5_ concentrations and regional effects on air quality and climate using the new CMIP6 scenarios. A total of 38 simulations were analyzed from nine CMIP6 climate models for historical and future SSP scenarios. The CMIP6 models, experiments, and methodology are described in Section 2, followed by an analysis of the model data in Section 3. Summaries and Discussions are presented in Section 4.

## 2. Data and Methodology

### 2.1. Simulation Data from CMIP6 Archive

In this study, we used 38 simulations from nine CMIP6 models (Table 2) for the first realizations that were available at the time of this study (up to February 2021) from the Earth System Grid Federation (ESGF). All the model outputs were bilinearly interpolated onto a grid with a horizontal resolution of 1.875° × 1.25°, which is also used by the UK Earth System Model (UKESM1-0-LL) [44]. The bilinear interpolation method tends to underestimate local maxima but does not create fictitious local peaks. The multimodel ensemble (MME) was examined by using the arithmetic mean of the model output, with the same weight assigned to each model. Six different experiments were selected for each CMIP6 model: historical simulation (Historical) was forced by the observed history of anthropogenic sources and natural forcing trends over a 164-year period, from 1850 to 2014.

**Figure 1:**
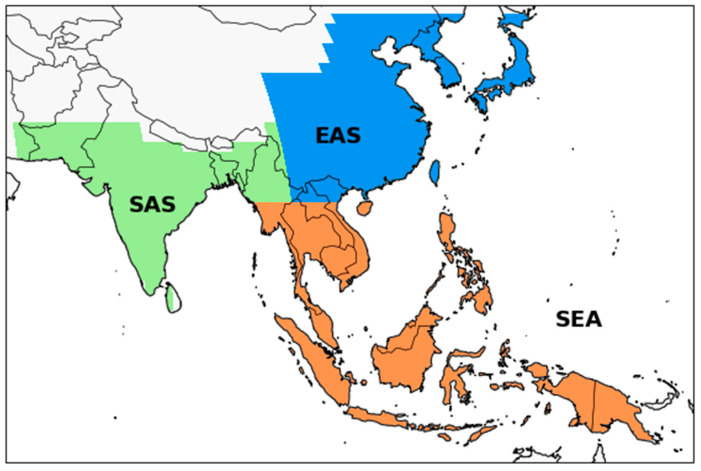
Three subregional Asian domains analyzed in this study, adopted from Iturbide et al. (2020) [53]. Shaded colors indicate three major regions: East Asia (blue; EAS), South Asia (green; SAS), and Southeast Asia (orange; SEA).

The future changes in air pollutants were then projected under different SSP scenarios throughout the 21st century (Table 3). For CMIP6, the four SSPs were categorized as SSP1–2.6 (SSP126), SSP2–4.5 (SSP245), SSP3–7.0 (SSP370), and SSP5–8.5 (SSP585) for the period from 2015 to 2100. O’Neill et al. [37] provide a more detailed description of the CMIP6 simulation design for the future scenarios. CMIP6 model data for the SSP3-7.0-lowNTCF scenario (SSP370 with cleaner air-quality policies; SSP370-lowNTCF) over the 2015–2055 period, derived from AerChemMIP [30], was also used in this study. The baseline historical simulation period of 1995–2014 was selected as present day (PD) in this study, whereas the near-future period of 2021–2040 and mid-future period of 2041–2060 were selected for the analysis of future changes in air pollution. Overall, regional analyses of three Asian subregions (Figure 1) were selected for this study, following the domain of Iturbide et al. (2020) [53] prepared for the IPCC sixth assessment report (AR6).

### 2.2. Satellite Data

This study used satellite-derived global annual mean surface PM_2.5_ concentration grid data from the NASA Socioeconomic Data and Application Center (SEDAC) for evaluating the simulated surface PM_2.5_ concentrations. These data were estimated using a combined geophysical–statistical method with information from aerosol optical depth (AOD) retrievals from the NASA Moderate Resolution Imaging Spectro Radiometer (MODIS), Multiangle Imaging Spectro Radiometer (MISR), and the Sea-Viewing Wide Field-of-View Sensor (SeaWiFS) instruments with the GEOS–Chem chemical transport model. The data were then calibrated to global ground-based measurements of PM_2.5_ concentrations for large-scale health and environmental research [54,55]. This high-resolution (0.01° × 0.01°) dataset is provided from the SEDAC website (available at https://sedac.ciesin.columbia.edu (accessed on 5 April 2021)) and covers the global land surface from 70° north to 55° south over the 1998–2016 period. Surface PM_2.5_ observational data were re-gridded onto the same resolution as the CMIP6 multimodel ensemble product (1.875° × 1.25°) for evaluation purposes. We focused on the 1998–2014 period to match the present-day (1995–2014) CMIP6 historical simulations.

### 2.3. Methodology

This study explored the impact of fine particulate matter on the air quality in Asia using SSP scenarios from the CMIP6 archive. However, not enough models provided a direct calculation of PM_2.5_ concentrations and as such we had to use an approximation that accounts for all aerosol components that were consistent across all CMIP6 models. Therefore, we calculated the PM_2.5_ concentrations using the offline method, as shown in Equation (1). Here, BC, OC, NH_4_, SO_4_, NO_3_, DU, and SS represented black carbon (BC), total organic carbon (OC), ammonium (NH_4_), sulfate (SO_4_), nitrate (NO_3_), dust (DU), and sea-salt (SS) particles, respectively, from the lowest model level in the CMIP6 individual model following previous studies [21,26,28,33,35,56]. The factors 0.1 and 0.25 indicated the DU and SS PM_2.5_ size fraction. Note that only a few CMIP6 models include the simulation of ammonium and nitrate particles in their aerosol-chemistry schemes (CESM2-WACCM model included NH4 particle and the GFDL-ESM4, GISS-E2-1G models included both NH4 and NO3). The analysis area was divided into three subdomains: East Asia (EAS), South Asia (SAS), and South East Asia (SEA) (Figure 1). The analyses of the regional annual mean PM_2.5_ gridded data were conducted based on a multimodel ensemble (MME).

Estimated PM_2.5_ = BC + OC + NH_4_ + SO_4_ + NO_3_ + (0.1 × DU) + (0.25 × SS) (1)

This study also attempted to estimate the effects of long-term exposure to fine particulate matter using the annual mean surface PM_2.5_ concentrations calculated from Equation (1). The air-quality levels in the six categories were classified based on the annual mean PM_2.5_ from the WHO air-quality guidelines and interim targets (Table 4), namely the AQG (air-quality guideline), IT-3 (interim target 3), IT-2 (interim target 2), IT-1 (interim target 1), OT (over target), and ST (significant target). The higher AQI values indicated a greater level of air pollution and therefore pose a higher risk to health, which could lead to the premature death of residents. For example, an AQI value of ‘AQG’ denoted a good air-quality level with little or no potential to affect public health. However, an AQI value of IT-1 suggests that air pollution may have contributed to an approximately 15% higher premature mortality risk relative to the AQG level [57]. Therefore, the WHO guidelines for outdoor particulate matter recommend that the annual average PM_2.5_ does not exceed 10 µg/m^3^. In this study, changes in the annual mean surface PM_2.5_ concentrations and AQI index, and their effects on regional future climates were analyzed according to the SSP scenarios from the CMIP6 simulated data.

## 3. Results

### 3.1. Evaluating the Estimated PM_2.5_ from CMIP6 Models in the Present–Day Period

**Figure 2:**
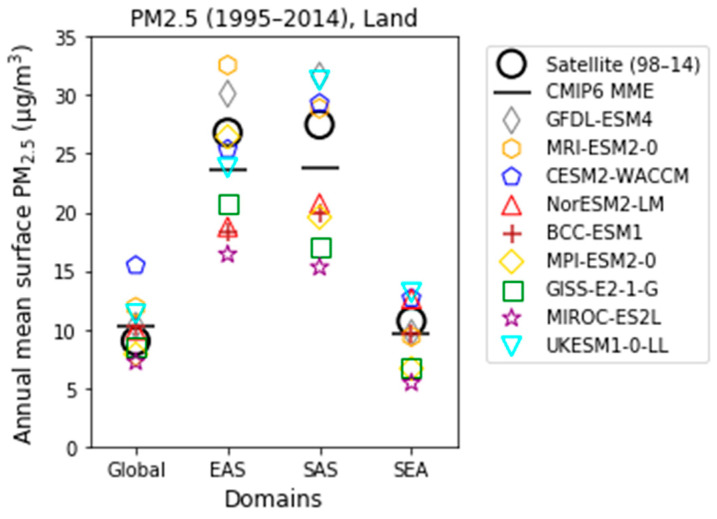
Comparison of the domain averaged annual mean surface PM_2.5_ concentrations from satellite data and CMIP6 historical simulations. The black circles represent the satellite data (1998–2014) and the remaining symbols indicate data obtained from the CMIP6 historical simulations for the present day (1995–2014). The thick horizontal black lines represent the ensemble mean of the CMIP6 models. Domain regions are defined in Figure 1.

The performance of fine particulate matter was evaluated from the nine CMIP6 models (Table 2). The annual mean surface PM_2.5_ concentrations for the globe and for the EAS, SAS, and SEA domain regions were estimated using historical simulations in the individual CMIP6 model and MME, and the results were compared with the available satellite-based PM_2.5_ data for validation. The CMIP6 MME mean PM_2.5_ (black horizontal line in Figure 2) showed good agreement with the satellite-derived PM_2.5_ (black open circle in Figure 2) for the present-day period. For both the CMIP6 model-simulated and satellite-derived data, annual mean surface PM_2.5_ concentrations in EAS and SAS were more than twice as high than worldwide and in the SEA region. The CMIP6 MME for the present-day annual mean surface PM_2.5_ was 9.6 ± 2.5 µg/m^3^ globally, 23.6 ± 5.3 µg/m^3^ in EAS, 23.7 ± 6.2 µg/m^3^ in SAS, and 9.6 ± 2.8 µg/m^3^ in SEA. The simulated PM_2.5_ concentrations in EAS and SAS were marginally underestimated compared to the satellite data. This might be because only a few CMIP6 models account for nitrate aerosols as described in Section 2. And the deviation between the individual CMIP6 models was also greater than those of the global and SEA regions. The larger model diversity over the EAS and SAS regions was consistent with a previous study [33] that demonstrated that the inter-model differences might be attributed to different simulations of historical changes in the anthropogenic aerosol components.

**Figure 3:**
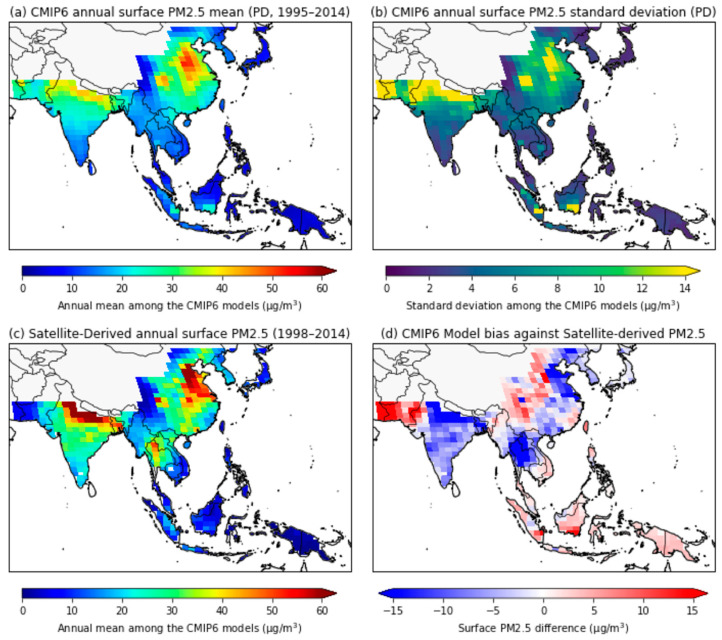
The spatial distribution of annual mean surface PM_2.5_ concentrations from the CMIP6 historical simulations for the present–day period (1995–2014) and satellite data (1998–2014). (**a**) Multimodel mean, (**b**) standard deviation of the multimodel mean, (**c**) satellite–derived annual surface PM_2.5_ concentrations, and (**d**) the difference between multimodel mean and satellite data.

The regional spatial distribution of the annual mean PM_2.5_, estimated based on the CMIP6 MME, was compared to satellite-derived PM_2.5_ concentrations for the present day (Figure 3). The results indicate that the industrialized and highly populated countries of China and India are exposed to the highest regional PM_2.5_ concentrations. For this reason, the annual mean concentration of PM_2.5_ in recent decades was the highest in the Beijing and Zhangzhou regions of central China, with values exceeding 50 µg/m^3^ (Figure 3a). Moreover, significant variation was simulated between the CMIP6 individual models in areas with large anthropogenic emission sources, such as Eastern China and Northern India (Figure 3b). Conversely, the MME PM_2.5_ mean and standard deviation was relatively low in southern India, Japan, and Indonesia. Overall, the PM_2.5_ concentrations in most of the regions analyzed in this study, except for Japan, the Philippines, and eastern Indonesia, exceeded the WHO air-quality guidelines (10 µg/m^3^ annual mean).

In this study, we adopted the CMIP6 MME analysis to understand the uncertainty due to differences in the physical process of various climate models. The spatial distribution of the annual mean PM_2.5_ for the present-day period, calculated from CMIP6 historical simulations, showed similar distribution patterns to those of satellite-derived PM_2.5_ data (Figure 3a,c). However, the simulated-PM_2.5_ concentrations were underestimated compared to the satellite-derived PM_2.5_ concentrations obtained for eastern China, northern India, and Thailand (Figure 3d). Conversely, for western China, Pakistan, and Indonesia, the CMIP6 model concentrations were overestimated relative to the satellite data. Moreover, the diversity between the CMIP6 models was large over eastern China and northern India, which experience high levels of PM_2.5_. Despite model diversity and regional biases (Figure 3b,d), the simulated PM_2.5_ concentrations from the CMIP6 MME showed reasonable domain-averaged values in the present-day climate compared to the satellite-derived PM_2.5_ concentrations (Figure 2). Therefore, the historical CMIP6 MME simulations were used as a reference period (1995–2014) for near- and mid-future climate changes for the remainder of this study.

### 3.2. Future Changes in Simulated PM_2.5_ Concentrations and the Air-Quality Index

**Figure 4:**
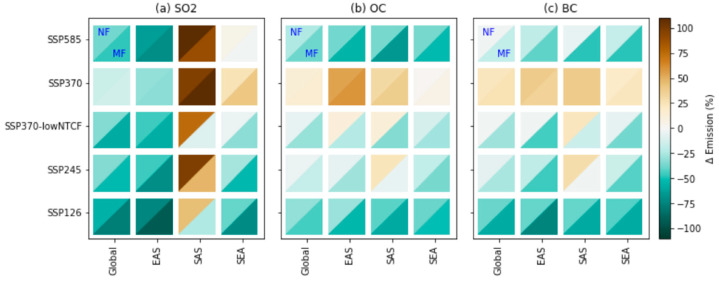
Future changes (unit: %) in the near future (2021–2040, left-top triangle; NF) and mid-future (2041–2060, right–bottom triangle; MF) relative to the present day (1995–2014) for annual mean total emissions of (**a**) SO_2_, (**b**) OC, and (**c**) BC worldwide and for the Asian regions (EAS, SAS, and SEA) analyzed in this study under the various CMIP6 SSP scenarios.

Figure 4 shows the projected global and regional changes in primary aerosols and their precursor emissions relative to the present day. This data was used as the input data for the CMIP6 models. Anthropogenic aerosol and precursor (organic carbon, black carbon, and sulfur dioxide) emissions used in each CMIP6 model were obtained from the same dataset. It is worth noting that the emissions of natural aerosol sources such as dust and sea-salt are different, depending on the physical configuration of the individual CMIP6 model (not shown).

Overall, except for the SSP3-7.0 scenario, the emissions of anthropogenic aerosols and precursors for EAS, SEA, and worldwide showed a decreasing tendency in the near and mid-future. In the SAS region, SO_2_ showed an increasing trend (up to 50%) in all the near-future scenarios and a continuous increase in the mid-future for the SSP5-8.5, SSP3-7.0, and SSP2-4.5 scenarios (Figure 4a). In addition, future OC and BC emissions in SAS are also expected to increase in the near future (Figure 4b,c) for all the scenarios except SSP1-2.6 and SSP5-8.5, which include the rapid implementation of air pollution controls. For the SSP3-7.0 scenario, which specifies inadequate air pollution controls, anthropogenic aerosols and precursor emissions are expected to increase or remain at the present-day levels in all regions.

**Figure 5:**
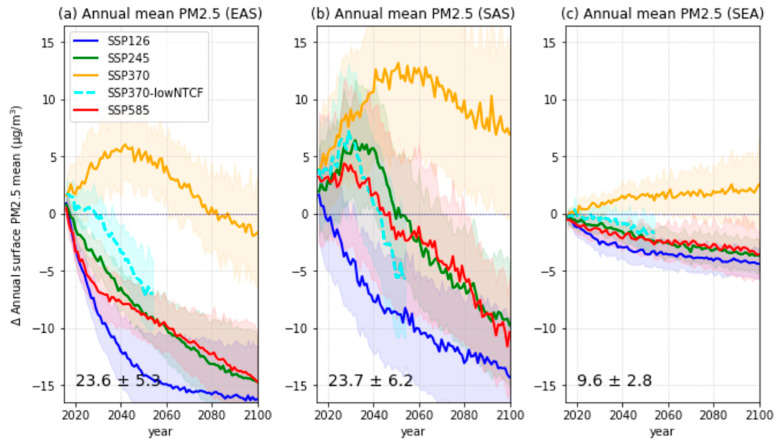
Future changes in the annual surface mean PM_2.5_ concentrations relative to the present-day (1995–2014) mean for various CMIP6 SSP scenarios for the three Asian regions: (**a**) EAS, (**b**) SAS, and (**c**) SEA. Each line represents the MME mean, and the shaded areas represent a ±1 standard deviation of the mean. The MME regional mean value and ±1 standard deviation for the present day is indicated in the bottom left corner.

Future changes in the annual mean surface PM_2.5_ concentrations in the Asian regions were examined using the different CMIP6 SSP scenarios (Figure 5). For the significant air-quality control scenarios, SSP1-2.6 and SSP5-8.5, a considerable decrease in the annual mean surface PM2.5 was predicted for the near future in EAS (Figure 5a). The results indicated a decrease in PM2.5 of more than 50% by the end of the 21st century compared to present-day levels. These changes are driven by the large emission controls of anthropogenic aerosols and their precursors (Figure 4). The decreasing trend in the SSP2-4.5 scenario was similar to that in the SSP5-8.5 scenario; however, higher PM2.5 concentrations were simulated in the near-future period. In the SSP3-7.0 scenario, which includes weak air-quality controls, the annual mean PM2.5 concentrations increased until the mid-21st century, and then subsequently decreased to levels similar to the present day by the end of the 21st century.

In the SAS region, future changes of the annual mean PM_2.5_ in the SSP1-2.6 and SSP3-7.0 scenarios were similar to those in EAS. However, significantly large diversities were simulated between the CMIP6 models (Figure 5b), particularly for the far future. Moreover, unlike the predictions for EAS, changes in the annual mean PM_2.5_ for the SSP2-4.5 and SSP5-8.5 scenarios were characterized by a marginal increase in the near-future period (green and red solid lines in Figure 5b). It was also found that the future changes in PM_2.5_ concentrations in SAS exceeded those in EAS, particularly for the SSP3-7.0 scenario, which includes the weak implementation of air-quality controls. In the SEA region, both the changes in the annual mean PM_2.5_ and the model diversity were smaller than in the EAS and SAS regions (Figure 5c). However, a continuous increase (approximately 30% in the far future) in the PM_2.5_ in the SEA region was found for the SSP3-7.0 scenario, whereas the PM_2.5_ concentrations simulated for the EAS and SAS regions decreased after the mid-future period.

**Figure 6:**
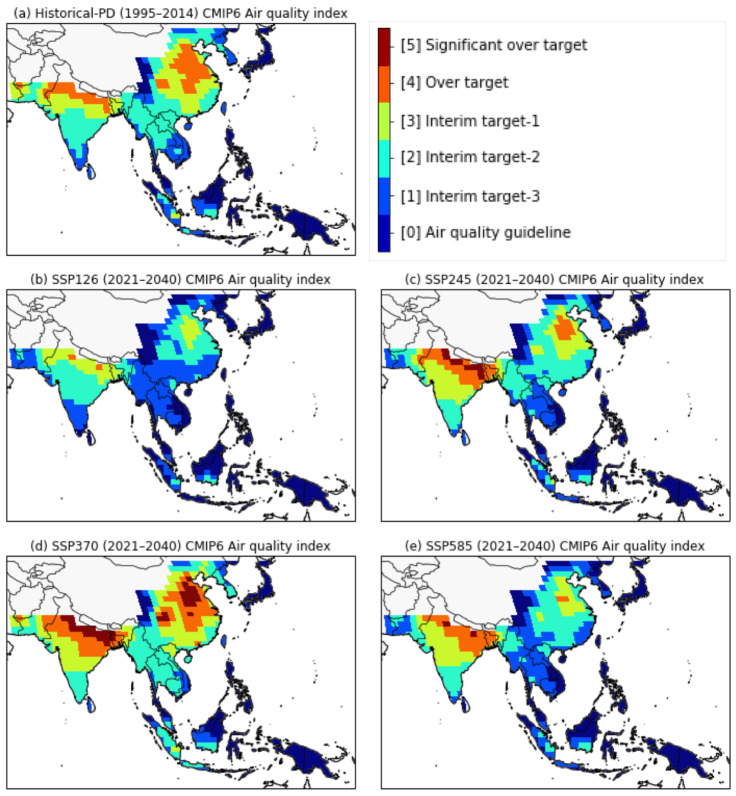
Spatial distribution of AQI based on WHO air-quality guidelines and interim targets for long-term exposure of PM_2.5_ for (**a**) the present day from CMIP6 historical simulations, and for the near future from the (**b**) SSP1-2.6, (**c**) SSP2-4.5, (**d**) SSP3-7.0, and (**e**) SSP5-8.5 scenarios. AQI value classifications are defined in Table 4.

In this study, we calculated the AQI index for the present-day and near-future periods using the simulated annual surface mean PM_2.5_ concentrations obtained from the CMIP6 MME mean (Figure 6). Higher AQI values are an indicator of increasing long-term exposure to PM_2.5_, which could pose a severe threat to human health and increase the risk of premature death (Table 4). The present-day AQI values for the historical simulations in the EAS and SAS regions were typically above the level of ’interim target 2′ (the cyan-colored areas in Figure 6a). In particular, the AQI values over northern India and eastern China, around mega cities such as Beijing, Shanghai, Delhi, and Kolkata were at ‘over target’ level (the orange-colored area in Figure 6a). Based on the simulated PM_2.5_ concentrations for the present day, we determined that the PM_2.5_ levels for Japan, western China, and eastern SEA, situated far from air pollution source regions, were below the specified WHO AQG annual mean level of 10 µg/m^3^ (the indigo-colored area in Figure 6a). These results are consistent with a WHO report published in 2016 on annual mean PM_2.5_ levels in the relevant Asian regions [2].

**Figure 7:**
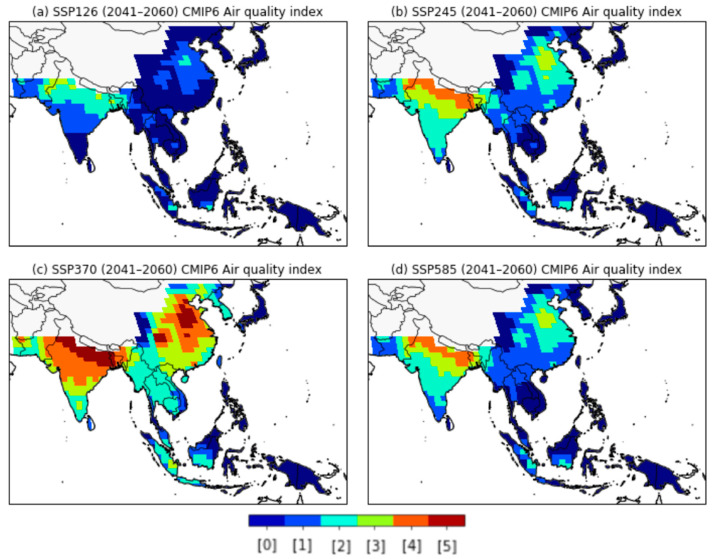
Spatial distribution of AQI based on WHO air-quality guidelines and interim targets for long-term exposure of PM_2.5_ for the mid-future (2041-2060) from the (**a**) SSP1-2.6, (**b**) SSP2-4.5, (**c**) SSP3-7.0, and (**d**) SSP5-8.5 scenarios. AQI value classifications are defined in Table 4.

In the SSP1-2.6 scenario, the AQI values in EAS are expected to improve substantially in the near future. In particular, the AQI level in China was lowered to the interim target range, and the AQI values for the Korean peninsula were below the specified WHO AQG levels (Figure 6b). The ‘over target’ area in northern SAS also showed a decrease compared to present-day levels. The spatial distributions of the AQI values showed similar patterns for the SSP2-4.5 and SSP5-8.5 scenarios, with AQI levels showing slightly improvement in EAS and worse in SAS compared to the present day (Figure 6c,e). These results were attributed to increases in the annual mean PM_2.5_ concentrations in SAS due to higher anthropogenic aerosol and precursor concentrations in the near-future period (Figure 4 and Figure 5b). In the SSP3-7.0 scenario, the AQI values were expected to increase significantly in EAS, SAS, and northern SEA (Figure 6d). In particular, the AQI values over the northern SAS and eastern EAS regions were calculated as ‘significantly over target’ (the red-colored area in Figure 6d). We also estimated resident population in areas exceeding the WHO interim target using the total population dataset from the Inter-Sectoral Impact Model Inter-Comparison Project phase 2b (ISIMIP2b) [59]. In the SSP3 scenario, more than 1.6 billion people (around 1 billion in SAS and around 0.6 billion in EAS) will be exposed to air pollution exceeding the IT levels of AQI in the near future. These changes of AQI values in the mid-future are much more pronounced than for the near future (Figure 7). In the SSP1-2.6 scenario, the AQI values in EAS, excluding inland China, were expected to be IT-3 (#1) or the lowest levels (AQG, #0) of AQI for the mid-future period. Conversely, in the SSP3-7.0 scenario, it was found that the AQI values in more than 50% of SAS regions were OT or ST levels.

**Figure 8:**
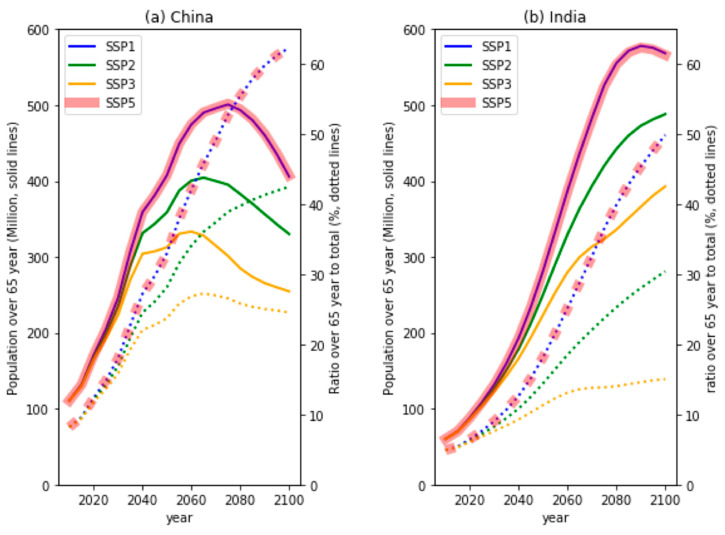
Time variation of total population (unit: million, solid lines) and ratio over 65 years to total population (unit: %, dotted lines) for the different SSP scenarios across (**a**) China and (**b**) India. Datasets were obtained from the International Institute for Applied System Analysis (IIASA) [39].

The annual mean PM_2.5_ concentrations and AQI levels in EAS were shown to improve overall in the future with the implementation of air-quality controls (Figure 5 and Figure 6). However, the predicted AQI values remained above the level of ‘interim target 2′ and ‘interim target 1′ in eastern China. Moreover, our results indicated that the AQI values for the northern region of SAS in the near future will exceed present-day levels. CMIP6 models simulated that most regions over China and India in the near-future period will be exposed to levels of PM_2.5_ concentrations that exceed the WHO air-quality guideline values. In addition, the proportion of populations over the age of 65 years old in China and India is projected to increase significantly under the SSP scenarios (Figure 8). This ratio could be as high as 35% by 2050, then increasing to up to 60% by 2100 (Figure 8a). Rapid population ageing coupled with AQI levels higher than the WHO guidelines in the future may increase the risk of premature mortality from the long-term exposure to PM_2.5_.

### 3.3. Regional Response to Future Air Pollution Mitigation

**Figure 9:**
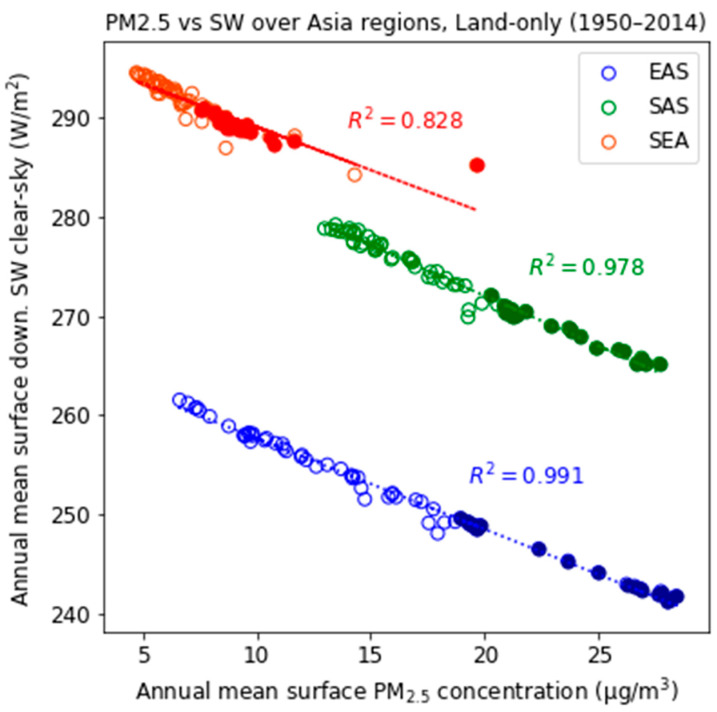
Scatter plot and regression line of simulated annual mean surface PM_2.5_ concentrations and clear-sky surface downwelling shortwave radiative fluxes from 1950 to 2014 for the Asian regions. The closed circles represent the ensemble mean values for the present-day period (1995–2014).

From the middle of the 20th century to the present day, the annual mean surface PM_2.5_ concentrations across Asia have increased considerably due to industrialization (Figure 9). The aerosol-radiative effects due to rapidly increasing anthropogenic aerosols have played a critical role in cooling in recent decades, partially offsetting the GHGs warming [13,60,61]. Aerosol can also affect the SW indirectly by altering cloud properties such as albedo and lifetime, but have more uncertain effects. For this reason, regional PM_2.5_ concentrations in CMIP6 historical simulations are closely correlated with changes in the clear-sky surface downwelling shortwave radiative flux (SW). Particularly, clear-sky SW in EAS showed a significant negative trend, decreasing from 260 to 240 Wm^−2^ (blue circles in Figure 9). Therefore, the implementation of air pollution controls can accelerate global and regional warming by recovering reduced surface radiation in the present day. According to previous study [62], the warming in response to reduced anthropogenic aerosols in China is likely already occurring recently.

**Figure 10:**
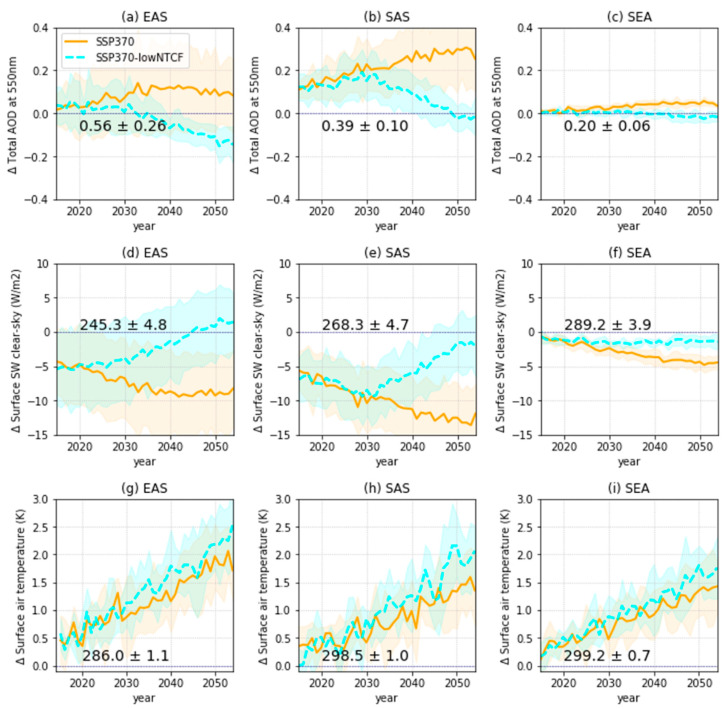
Future changes in annual mean total aerosol optical depths (**a**–**c**), clear-sky surface downwelling shortwave fluxes (**d**–**f**), and surface air temperatures (**g**–**i**) relative to the present-day (1995–2014) mean for the SSP3-7.0 and SSP3-7.0-lowNTCF scenarios for the three Asian regions (EAS, SAS, and SEA). Each line represents the MME mean and the shading represents the ±1 standard deviation of the mean. The MME regional mean value and ±1 standard deviation for the present day is indicated near the horizontal lines.

To understand the warming effects by anthropogenic aerosol reductions, we examined the differences between the weak air-quality control simulations (SSP3-7.0) and the strong air quality control simulations (SSP3-7.0-lowNTCF), which assumes the implementation of stringent air-quality control policies under the same conditions as the SSP3-7.0 scenario (Table 3, Figure 10). The significant difference in annual mean PM_2.5_ concentrations between the SSP3-7.0 and SSP3-7.0-lowNTCF (the orange and cyan lines in Figure 5) scenarios contributed to changes in the total aerosol optical depth (AOD) and clear-sky SW in the middle of the 21st century (Figure 10a–f). The regional AODs were reduced and the surface SW clear-sky increased considerably in SSP3-7.0-lowNTCF compared to SSP3-7.0 simulations. In particular, these changes in the EAS and SAS regions, which recorded high levels of PM_2.5_, showed relatively large differences compared to those in SEA. Moreover, warming trends (approximately +0.5 K in 2050) were simulated in response to increases in the surface SW clear-sky (Figure 10g–i). However, significant model diversities were found for the surface air temperature changes. Despite large model uncertainties of surface air temperature in all regions, implementations of strong air-quality controls seem to contribute to regional warming in the future. Therefore, reducing long-term exposure to PM_2.5_ can impact the rate of regional warming, so should be considered together when implementing climate change mitigation initiatives. Detailed warming mechanisms involved in climate sensitivity, aerosol-cloud interaction, and the temperature advections related to large-scale circulation require further research to understand CMIP6 model uncertainty; however, these factors are beyond the scope of this study.

## 4. Conclusions

This study performed an initial analysis of the long-term changes in PM_2.5_, AQI, and climate responses for three major regions in Asia under different future climate and air pollution control scenarios. The regional PM_2.5_ concentrations were estimated using simulations from nine CMIP6 models. The model performance of the estimated PM_2.5_ concentrations from historical simulations was evaluated against satellite-derived data from NASA’s Socioeconomic Data and Application Center. Elevated annual mean PM_2.5_ concentrations (>50 µg/m^3^) were simulated for the EAS and SAS regions, which are heavily industrialized and densely populated. Conversely, simulated present-day annual mean PM_2.5_ concentrations in SEA were relatively lower than in EAS and SAS. Overall, the simulated annual mean PM_2.5_ concentrations for all the analyzed regions except for Japan, the Philippines, and Indonesia exceeded the WHO’s recommended air-quality guidelines (10 µg/m^3^) for the present day. Moreover, a comparison of the estimated PM_2.5_ levels from the CMIP6 models with satellite data revealed an underestimation in the data for the three Asian domains, and particularly for EAS and SAS. Despite model diversity and regional biases, the simulated PM_2.5_ showed reasonable domain averaged values in the present day compared to the satellite-derived PM_2.5_ concentrations.

Future changes in the simulated annual mean PM_2.5_ were also examined for the various SSP scenarios. Rapid decreases in SSP1-2.6 and SSP5-8.5 for the scenarios assuming strict air-quality control were shown across the Asian regions in the near future. Unlike for EAS, the scenarios predicted an increase in PM_2.5_ concentrations in SAS in the near future. The changes in the annual mean value and model diversity for SEA were smaller than in the EAS and SAS regions. For the slowest deployment of air pollution controls (SSP3-7.0 scenario), annual mean PM_2.5_ showed an increase across all regions until the mid-21st century. We also attempted to understand the long-term exposure risk to PM_2.5_ using the AQI based on the WHO air-quality guidelines and interim targets. The present-day AQI level in EAS and SAS typically exceeded ‘interim target 2′, and several regions exceeded the range of the WHO interim target. In the SSP1-2.6, the CMIP6 models predicted a substantial decrease in AQI values in EAS and SAS. Similar trends were simulated under the SSP2-4.5 and SSP5-8.5 for EAS, whereas the AQI values in SAS were predicted to increase due to increasing PM_2.5_ levels in the near future. In the SSP3-7.0, the number of regions beyond interim target is expected to increase significantly. Moreover, the risk of premature mortality from long-term exposure to PM_2.5_ could significantly increase in China and India due to the rapid population ageing in the future.

This study is among a few climate-modeling studies that explored the impact of future air quality in Asia under the SSP scenarios. Overall, our results indicated that the CMIP6 climate model simulations successfully reproduced the regional surface PM_2.5_ concentrations for the present day. The results in this study were consistent with a previous study [33] that demonstrated that the decreasing (increasing) trends of simulated regional PM_2.5_ are related to the strong (weak) implementation of future air-quality controls. In addition, this study analyzed the AQIs based on the WHO guidelines for the three major Asian regions and investigated the regional impact of air pollution controls implementations on PM_2.5_ levels. An additional finding of this study was the potential acceleration of global warming in Asia with future decreases in anthropogenic aerosol emissions reducing the radiative cooling effect of aerosols.

Despite the significant findings of this study, several limitations were noted. We adopted the CMIP6 multimodel ensemble analysis to understand the impact of fine PM on regional air quality and their uncertainties. However, the estimated PM_2.5_ concentrations from the CMIP6 models were underestimated compared to the satellite-derived data. Only a few models consider ammonium nitrate as aerosol components in their schemes, which may be one of the reasons for underestimation of PM_2.5_ concentrations. In addition, the large model diversity may be attributed to the differences in the aerosol microphysical processes among the CMIP6 individual models. Detailed causes of model uncertainties should be explored in further studies; however, they are beyond the scope of this study. This study also emphasized the effects of premature mortality due to increasing PM_2.5_ concentrations, coinciding with a rapidly ageing population. However, a more detailed risk analysis with the available data from the CMIP6 simulations is required to verify this conclusion. These shortcomings will be addressed in our future research.

## Figures and Tables

**Table 1:** Shared socioeconomic pathways’ (SSPs) air pollution control levels (Based on [42]).

SSP Scenarios	Emission Factors
SSP1 and SSP5	Strong decrease (fastest and widest implementation of air pollution controls)
SSP2	Medium decrease (significant advancement in pollution control, yet less than in SSP1 and SSP5)
SSP3 and SSP4	Weak decrease (slowest deployment of air pollution controls)

**Table 2:** List of CMIP6 climate models used for historical and future scenario experiments in this study. Circles indicate that the model was used, but asterisks indicate a lack of surface PM_2.5_ concentration data. Detailed descriptions of the CMIP6 models are provided in Supplementary Table S1.

Model Name	Historical	SSP1–2.6	SSP2–4.5	SSP3–7.0	SSP3–7.0-lowNTCF	SSP5–8.5
UKESM1-0-LL [44]	○	○	○	○	○	○
GFDL-ESM4 [45]	○	○	○	○	○	○
NorESM2-LM [46]	○	○	○	○	○	○
GISS-E2-1-G [47]	○	○	○	○	○	○
MIROC-ES2L [48]	○	○	○	○	*	○
MRI-ESM2-0 [49]	○			○	*	
CESM2-WACCM [50]	○			○	○	
BCC-ESM1 [51]	○					
MPI-ESM1.2-HAM [52]	○					
Total number of models	9	5	5	7	7	5

**Table 3:** List of CMIP6 experiments used in this study.

Experiment	Information
Historical (1850–2014)	The historical simulations use forcing due to both the natural causes and human factors over the period 1850 to 2014. These simulations were used to evaluate model performance.
SSP1-2.6 (2015–2100)	This scenario represents the low end of the range of plausible future pathways, and depicts the best-case future scenario from a sustainability perspective.
SSP2-4.5 (2015–2100)	This scenario represents the medium part of the range of plausible future pathways.
SSP3-7.0 (2015–2100)	This scenario represents the medium to high end of plausible future pathways.
SSP3-7.0-lowNTCF (2015–2055)	This scenario represents the SSP3-7.0 scenario with the reduced near-term climate forcer (NTCF) emissions, including aerosols.
SSP5-8.5 (2015–2100)	This scenario represents the high end of plausible future pathways. SSP5 is the only SSP scenario with emissions high enough to produce the 8.5 Wm^−2^ level of forcing in the year 2100.

**Table 4:** List of CMIP6 experiments used in this study.

	Index	PM_2.5_ (µg/m^3^)	Basis for the Selected Level [57]
5	Significantly over target (ST)	53–	Defined as a concentration that exceeds 150% of the interim target-1 level.
4	Over target (OT)	35–53	Defined as a concentration higher than the interim target and less than 150% of the interim target-1 level.
3	Interim target 1 (IT-1)	25–35	Approximately 15% higher long-term mortality risk relative to the air-quality guideline level.
2	Interim target 2 (IT-2)	15–25	These levels lower the risk of premature mortality by approximately 6% relative to the IT-1 level.
1	Interim target 3 (IT-3)	10–15	These levels reduce the mortality risk by approximately 6% relative to the IT-2 level.
0	Air-Quality Guideline (AQG)	0–10	Lower end of the range of significant effects on survival in response to long-term exposure to PM_2.5_. [58]

## Data Availability

The data presented in this study are available on request from the corresponding author. Datasets from CMIP6 simulations are available through the Earth System Grid Federation (ESGF).

## Supplementary Materials

The following are available online at https://www.mdpi.com/article/10.3390/ijerph18136817/s1, Table S1: Description of CMIP6 models used in this study.
