# Supplementary material for: Regional Features of Long-Term Exposure to PM2.5 Air Quality over Asia under SSP Scenarios Based on CMIP6 Models
Source: Int J Environ Res Public Health. 2021 Jun 25;18(13):6817. doi: 10.3390/ijerph18136817 (PMC8297095; doi:10.3390/ijerph18136817)
Supplement: Supplementary file 1 [file ijerph-18-06817-s001.zip › ijerph-1251553-supplementary.pdf]

Table S1: Descriptions of the CMIP6 models used in this study [33]

Model Name	Horizontal Resolution	Vert. levels (top level)	Aerosol scheme	Aerosol Species	Chemistry scheme
UKESM1-0-LL	1.25° x 1.875°	L85 (85 km)	GLOMAP-Mode (Modal scheme, mass and number). Mass-based bin scheme used for dust	SO_4, BC, OM, sea salt in 5 log-normal modes and dust in 6 bins	UKCA coupled stratosphere-troposphere interactive photolysis
GFDL-ESM4	cubed-sphere (c96) grid, with ~100 km native resolution, re-gridded to 1.0° x 1.25°	L49 (0.01 hPa)	Bulk mass-based scheme. 5 size bins used for sea salt and dust.	NH_4, SO_4, NO_3, NH_4, BC, OM, sea salt, dust	Interactive stratosphere-troposphere
NorESM2-LM	1.9° x 2.5°	L32 (3.64 hPa)	OsloAero6	SO_4, BC, OM, sea salt, dust (log-normal modes)	Simplified chemistry for use in aerosol scheme. Other fields prescribed.
GISS-E2-1-G	2° x 2.5°	L40 (0.1 hPa)	OMA (one moment aerosol scheme – mass-based)	SO_4, NO_3, NH_4, BC, OM treated as externally mixed with prescribed and constant size distribution. Sea salt has two size classes. Sectional scheme for dust with 5 size bins that can be coated with SO_4 and NO_3 to increase solubility.	Coupled troposphere-stratosphere chemistry scheme. Modified Carbon Bond Mechanism 4 (CBM-4) chemical mechanism
MIROC-ES2L	2.813° x 2.813°	L40 (3.0 hPa)	SPRINTAS	SO_4, BC, OM, sea salt, and dust in lognormal size distributions. External mixing assumed for SO4, sea salt, and dust aerosols	Simplified chemistry for use with aerosol scheme
MRI-ESM2-0	MRIAGCM3.5: 1.125° x 1.125°, MASINGAR mk-2r4c: 1.875° x 1.875°, MRICCM2.1: 2.813° x 2.813°	L80 (0.01 hPa)	MASINGAR mk2r4c	Mass-based scheme with externally mixed size distributions. SO_4 (three categories), BC (hydrophilic and hydrophobic), OM (hydrophilic and hydrophobic), sea salt (10 size bins), and dust (10 size bins).	Chemistry Climate Model version 2.1 (MRICCM2.1) covering troposphere, stratosphere, and mesosphere
CESM2-WACCM	0.9° x 1.25°	L70 (6×10^{-6} hPa)	MAM4 (modal scheme, simulating mass and number concentrations) with VBS-SOA	SO_4, BC, OM (both primary and secondary), sea salt, and dust	MOZARTTSMLT1 covering troposphere, stratosphere, mesosphere, and lower thermosphere
BCC-ESM1	2.813° x 2.813°	L26 (2.914 hPa)	Mass-based aerosol scheme. Prescribed stratospheric aerosols.	SO_4, BC (hydrophilic and hydrophobic), OM (hydrophilic and hydrophobic, sea salt (4 size bins), and dust (4 size bins). No nucleation or coagulation of aerosols	CAM-Chem (based on MOZART). Tropospheric only chemistry
MPI-ESM1.2-HAM	1.875° x 1.875°	L47 (0.01 hPa)	HAM2.3 (Modal scheme, mass and number)	SO_4, BC, OM, sea salt, and dust in 7 lognormal modes	Simplified sulfur chemistry. Other fields prescribed.
